# Is Location Everything? Regulation of the Endothelial CCM Signaling Complex

**DOI:** 10.3389/fcvm.2022.954780

**Published:** 2022-07-11

**Authors:** Harsha Swamy, Angela J. Glading

**Affiliations:** Department of Pharmacology and Physiology, University of Rochester, Rochester, NY, United States

**Keywords:** CCM, vascular malformations, scaffolding protein, subcellular localization, signaling

## Abstract

Recent advances have steadily increased the number of proteins and pathways known to be involved in the development of cerebral cavernous malformation (CCM). Our ability to synthesize this information into a cohesive and accurate signaling model is limited, however, by significant gaps in our knowledge of how the core CCM proteins, whose loss of function drives development of CCM, are regulated. Here, we review what is known about the regulation of the three core CCM proteins, the scaffolds KRIT1, CCM2, and CCM3, with an emphasis on binding interactions and subcellular location, which frequently control scaffolding protein function. We highlight recent work that challenges the current model of CCM complex signaling and provide recommendations for future studies needed to address the large number of outstanding questions.

## Introduction

Cerebral cavernous malformation (CCM) is a disease characterized by the formation of microvascular lesions primarily in the brain. These lesions derive from highly proliferative endothelial cells with poor barrier function (1–3). A consequence of this perturbed endothelial behavior is the formation of large vascular “caverns” lacking surrounding mural cells and astrocytes, as well as altered extracellular matrices surrounding the endothelial cells (2, 4). CCM occurs in the general population at a rate of ~0.5% (5), and may be hereditary (familial CCM) or occur sporadically. A genetic component for the development of CCM was first described in 1995 (6, 7). Further study revealed this gene to encode the protein Krev-Interaction Trapped 1 [KRIT1, also called CCM1; (8–10)], which had been previously identified as a binding partner of the small GTPase Rap1 (11), making KRIT1 the first protein linked to CCM pathogenesis. In 1998 two other genetic components were found (12), and by the mid 2000s these proteins were identified: CCM2/malcavernin (13) and the apoptosis-related protein, CCM3/PDCD10 (14, 15). Loss of function mutations in any of these three genes is sufficient to induce CCM lesion development, and have also been found in some sporadic CCMs (16). Recent studies have discovered other genes involved in CCM development, i.e., PIK3CA (17) and Cdc42 (18), but mutations in KRIT1, CCM2, or CCM3 remain the most commonly identified genetic basis for CCM.

The three core CCM proteins (i.e., KRIT1, CCM2 and CCM3) can bind directly to each other under normal physiological conditions (19–21) forming what is referred to as the CCM signaling complex. All three CCM proteins are scaffolding proteins, and each member of this complex has a unique set of binding partners that allow it to affect a wide range of cellular functions. Based on studies in human tissues, cell culture, and animal models, the CCM complex appears to promote endothelial quiescence by stabilizing cell-cell contact, limiting inflammatory and angiogenic signaling, and constraining proliferation (2, 4, 22–25). These abilities have been strongly linked to the regulation of mitogen activated protein kinase kinase kinase 3 (MEKK3), which binds to CCM2 (26, 27). However, how CCM2 curbs the activation of MEKK3 and its downstream signaling has not been established, nor has it been shown how loss of KRIT1 or CCM3 lead to activation of MEKK3 in cells that still maintain CCM2 expression. Moreover, studies using KRIT1 or CCM2 deficient cell or animal models have shown highly similar phenotypes (28), but loss of CCM3 causes more severe and acute CCM development both in animal models and human patients (29, 30), suggesting that the pathophysiology and progression of CCM lesion development is a complex process that is influenced by the specific gene affected.

These questions lay bare a significant gap in our current knowledge, that is, what mechanisms regulate the function of the CCM proteins and the CCM complex? Scaffolding proteins, such as the CCM proteins, are a functionally defined set of proteins which are able to bring together (at a minimum) two proteins in a relatively stable conformation and promote signaling between these target proteins. Scaffolding proteins function to organize cellular signaling, making possible the specific and temporal regulation of the vast array of signaling information that cells must continuously process. Regulation of scaffolding proteins depends, to some extent, on their domain composition and on the pathways in which they operate. Notably, scaffolding proteins must be localized to the same subcellular compartment as their target proteins, and relatedly, can promote the localization of their targets to specific cellular locations. Thus, protein expression and alternative splicing and control of location are common features in the regulation of scaffolding proteins. In addition, the interaction of scaffolding proteins with their targets can be regulated by post-translational modification (phosphorylation, ubiquitination, etc.) as well as autoinhibitory interactions between domains of the scaffolding protein itself. Indeed, the ERM family of scaffolding proteins, to which KRIT1 is structurally similar, are regulated by a well-characterized mechanism involving the interaction of the N-terminal ERM associate domain with sequences in the C-terminal ERM associate domain (31). In order to fully understand CCM pathogenesis, we need to know how the CCM proteins individually, and CCM complex formation as a whole, are regulated. In this review, we will examine what is currently known about how KRIT1, CCM2, and CCM3 are regulated, with an emphasis on binding interactions and sub-cellular localization, and discuss how that regulation may affect the function of the CCM complex.

## Domain Structure and Binding Interactions of CCM Proteins

### Krev-Interaction Trapped 1, KRIT1

**Figure 1:**
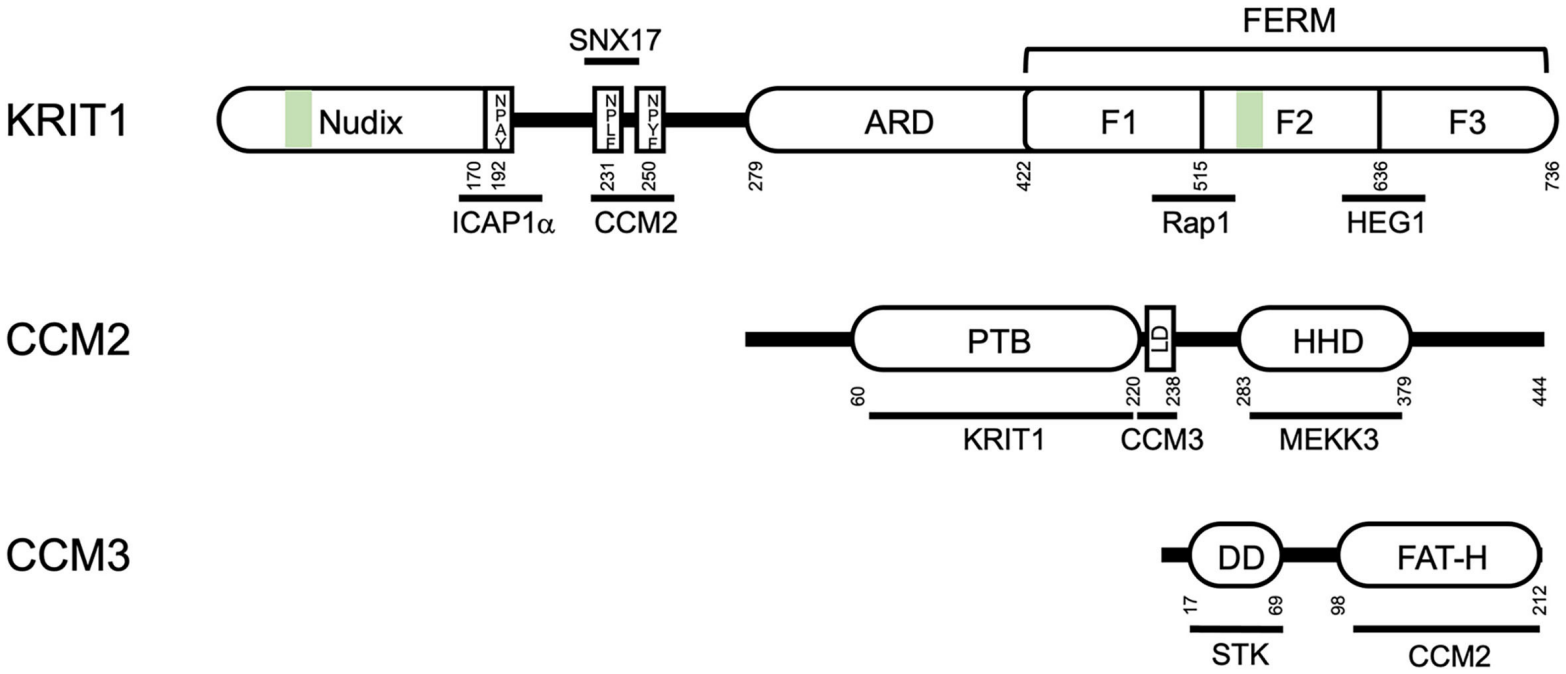
Domain structure of the CCM proteins KRIT1, CCM2 and CCM3. ARD- ankyrin repeat domain; FERM- Band 4.1, ezrin, radixin, moesin domain; F1, F2, and F3, globular subdomains of the FERM domain; PTB, protein tyrosine binding domain; LD, leucine-rich aspartate domain; HHD, harmonin homology domain; DD, dimerization domain; FAT-H, focal adhesion targeting domain; STK, sterile kinase. Green bars indicate nuclear localization sequences/microtubule binding sites. Residue numbers (from human proteins) are noted below the domain diagram.

KRIT1 is an 84kDa protein containing multiple protein-interacting domains (Figure 1). At the N-terminus (residues 1–170), Liu et al. identified a Nudix-like fold by structural homology (32). Nudix hydrolases are a superfamily of hydrolytic enzymes capable of cleaving nucleoside diphosphates, but the homologous domain in KRIT1 lacks catalytic activity. The remainder of the N-terminal half of KRIT1 is relatively unstructured, but contains three NPXY/F motifs (19, 32, 33) which are recognition sites for phospho-tyrosine binding (PTB) domains. The integrin regulatory protein ICAP1α binds to the first NPXY/F motif [NPAY, residues 192–195, (32)], while CCM2 is thought to bind to the second or third NPXY motifs (19, 34). The cytoplasmic sorting nexin adaptor protein sorting nexin 17 (SNX17) binds to the second NPXY motif (NPLF, residues 231–234). In the center of the protein are four ankyrin repeats [residues 259–422, (11, 35)] that putatively promote association with lipid membranes. The C-terminal half of KRIT1 is folded into a triple-lobed Band 4.1, ezrin, radixin, moesin (FERM) domain, which contains 3 subdomains (F1, F2, and F3) featuring a ubiquitin-like fold, a four-helix bundle, and a phospho-tyrosine binding domain, respectively (35–38). Co-crystallization of KRIT1 with the small GTPase Rap1 demonstrated that Rap1 binds to KRIT1 *via* an interaction with both the F1 and F2 subdomains (36), whereas the transmembrane orphan receptor Heart of Glass (HEG1) binds to an interface involving the FERM F1 and F3 subdomains (39). The C-terminal PTB domain (F3) of KRIT1 could theoretically interact with several NPXY-containing proteins, however the only defined interaction of this domain is an intermolecular interaction with a NPXY motif of KRIT1 itself. This interaction, between the C-terminal PTB domain and the first NPXY motif (40) is highly similar to the autoinhibitory self-interaction seen in other ERM family proteins (31). In addition, KRIT1 contains a nuclear localization sequence [NLS, residues 46–51, (41–44)], which may also be important for binding of KRIT1 to microtubules. Finally, while KRIT1 contains several predicted nuclear export sequences (43, 44), none have been confirmed to regulate subcellular trafficking of KRIT1.

### CCM2

CCM2 is a 49 kDa protein that contains a N-terminal PTB domain (13), which binds to KRIT1 (19, 44) and the cell death receptor TrkA (45) (Figure 1). A single point mutation in the CCM2 PTB, F217A, blocks the interaction of KRIT1 and CCM2 and is sufficient to cause CCM (21). CCM2 also contains a leucine-rich aspartate (LD)-like domain (residues 223–238), which binds to CCM3, and a C-terminal harmonin-homology domain (HHD) (residues 283–379), which is structurally similar to the N-terminal domain of the Usher syndrome protein harmonin (46). Interestingly, harmonin binds directly to the cell adhesion protein cadherin 23, expressed specifically in neurosensory epithelial cells, via two domains: its N-terminal domain and a PDZ domain (47). However, direct interaction of CCM2 with cadherins has not been reported. CCM2 also binds to the respective upstream kinases mitogen-activated protein kinase kinase 3 (MKK3) and MEKK3 (26, 48). One of the first studies of CCM2 identified it as an osmo-sensing scaffold for the MAP kinase MKK3 (48), a key mediator of p38 inflammatory signaling (49). Later studies demonstrated that CCM2 was a regulator of MEKK3, an upstream activator of big mitogen-activated protein kinase/extracellular signal regulated kinase 5 (BMK1/ERK5). Destabilization of the complex with MEKK3 through loss of any of the CCM proteins is a potent driver of CCM through perturbations of several pathways (26).

### CCM3

CCM3 (25kDa) is the most recently identified member of the CCM complex, and binds directly to CCM2 (Figure 1). CCM3 contains an N-terminal dimerization domain (50) which mediates interactions with the germinal center kinase III group of protein kinases, including sterile-kinases 24 and 25, forming part of the striatin interacting phosphatase and kinase (STRIPAK) signaling complex (51). CCM3 also contains a C-terminal focal adhesion targeting-homology (FAT-H) domain (50). Sequences within the FAT-H domain bind to the LD-like domain of CCM2 (21, 52), and also mediate interaction with the focal adhesion protein paxillin (50, 53).

## Regulation of CCM Protein Localization

**Figure 2:**
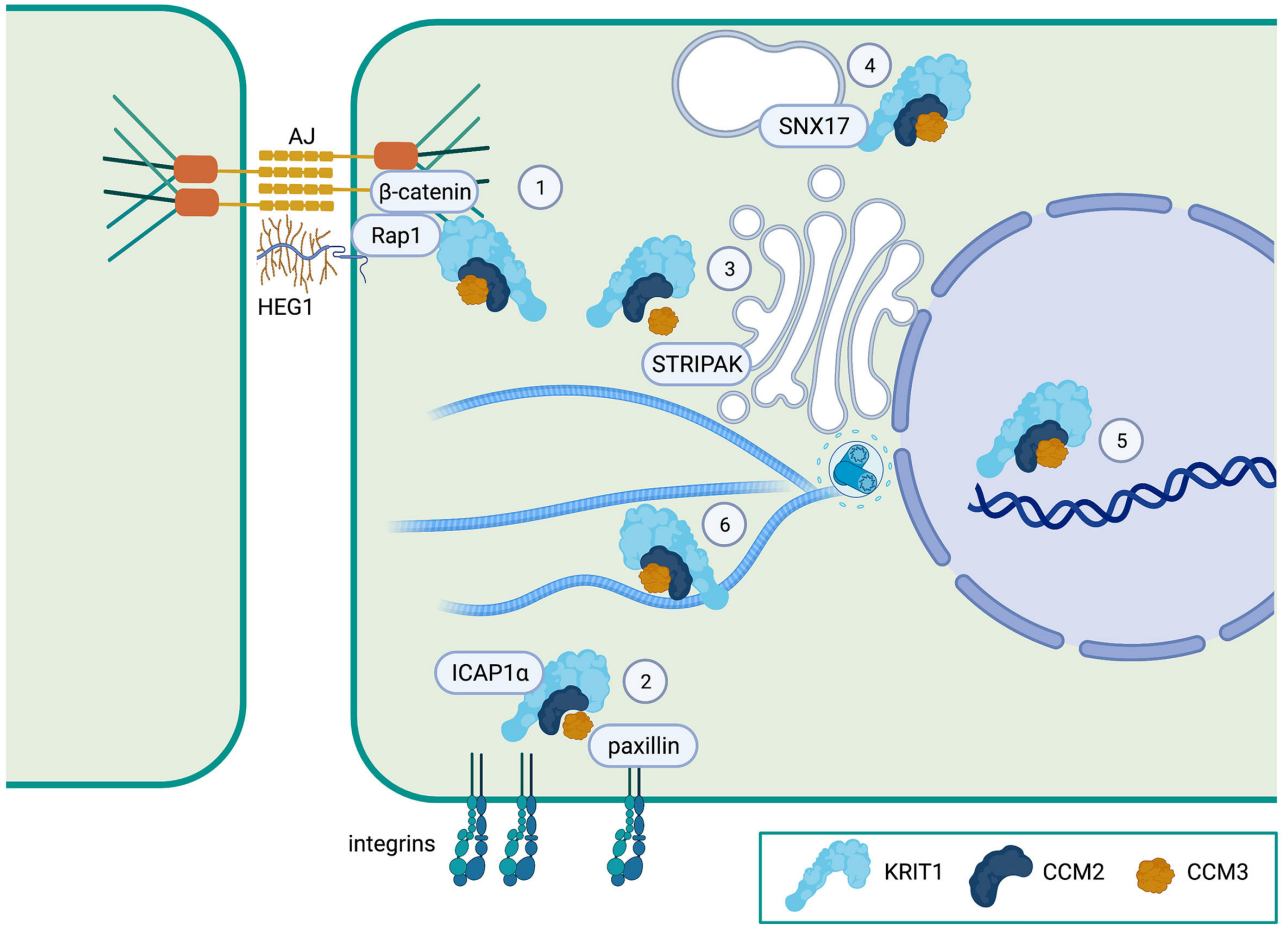
Proposed localization of the CCM complex. (1) The CCM complex is known to co-localize with β-catenin at adherens junctions (AJ) and to associate with the Heart of Glass (HEG1) orphan transmembrane receptor at the plasma membrane. (2) The CCM complex (at least KRIT1), regulates ICAP1α interactions with integrins. CCM3 can also bind to paxillin, a focal adhesion protein. (3) CCM3 is a member of the STRIPAK signaling complex which regulates cell polarity and Golgi assembly. (4) KRIT1 can bind to the endosomal trafficking protein sorting nexin-17 (SNX17). (5) All three CCM proteins can localize to the nucleus, but whether they have a nuclear function is unknown. (6) The CCM complex is also distributed in the cytoplasm, where KRIT1 can bind to microtubules. Created with Biorender.com.

All three CCM proteins have been shown to localize to the plasma membrane (particularly at cell-cell contacts), the cytoplasm, and the nucleus (Figure 2). While several studies have investigated the formation of the tripartite complex using co-immunoprecipitation, few have examined complex formation at the subcellular level. However, what evidence there is suggests that KRIT1∙CCM2 and CCM2∙CCM3 interactions can occur at or near the plasma membrane (50, 54). Alternatively, it is possible that some or all of the CCM proteins could function individually in unique locations. For example, CCM3, which associates with the STRIPAK complex at the Golgi (55) (Figure 2), also has been found at the apical epithelial membrane during excretory canal development in C. elegans (56), and in focal adhesions in cancer associated fibroblasts, where it regulates integrin-dependent adhesion and mechano-transduction (53). The relevance of these interactions to CCM pathogenesis or, more specifically, endothelial/epithelial barrier function is unknown. Indeed, only the KRIT1∙CCM2 association has been directly implicated in the stabilization of endothelial barrier function, as a point mutation of the PTB domain of CCM2 (F217A) results in a primarily cytoplasmic distribution of both proteins and loss of barrier function (54). Immunofluorescence imaging has also shown that KRIT1 and CCM2 colocalize at cell peripheries in COS-7 cells after osmotic shock (34), suggesting that the localization of this complex could be regulated by external signals. Consequently, subcellular localization is expected to play a key role in the regulation of CCM proteins and the function of the CCM complex, thus it is critical that we understand the mechanisms involved. In the next sections, we will review what is known about how the localization of CCM proteins are regulated and how that relates to the function of the CCM complex.

### CCM Complex Localization to Cell-Cell Contacts

Several studies have associated the localization of the CCM complex to sites of cell-cell contact with the ability of this complex to stabilize endothelial barrier function, suggesting that subcellular localization is critical to the functional consequence of active CCM complex signaling. Indeed, several mechanisms have been suggested to regulate localization of the CCM complex to the plasma membrane in general, and to adherens junctions specifically.

*In vitro* binding assays have shown that KRIT1, CCM2, and CCM3 can directly interact with cellular membranes. KRIT1 can bind phosphatidylinositol (4,5) bisphosphate (PIP2) *via* its FERM domain [residues 208–736, (40)], CCM2 preferentially interacts with phosphatidylinositol monophosphates (52), and CCM3 binds phosphatidylinositol (3,4,5) triphosphate [PIP3, (57)], potentially indicating that all CCM proteins can associate directly with membranes. While these direct interactions support the ability of the CCM complex to localize with membranes enriched in specific phospholipids, it has not been determined whether these interactions are sufficient for membrane localization of the complex.

**Figure 3:**
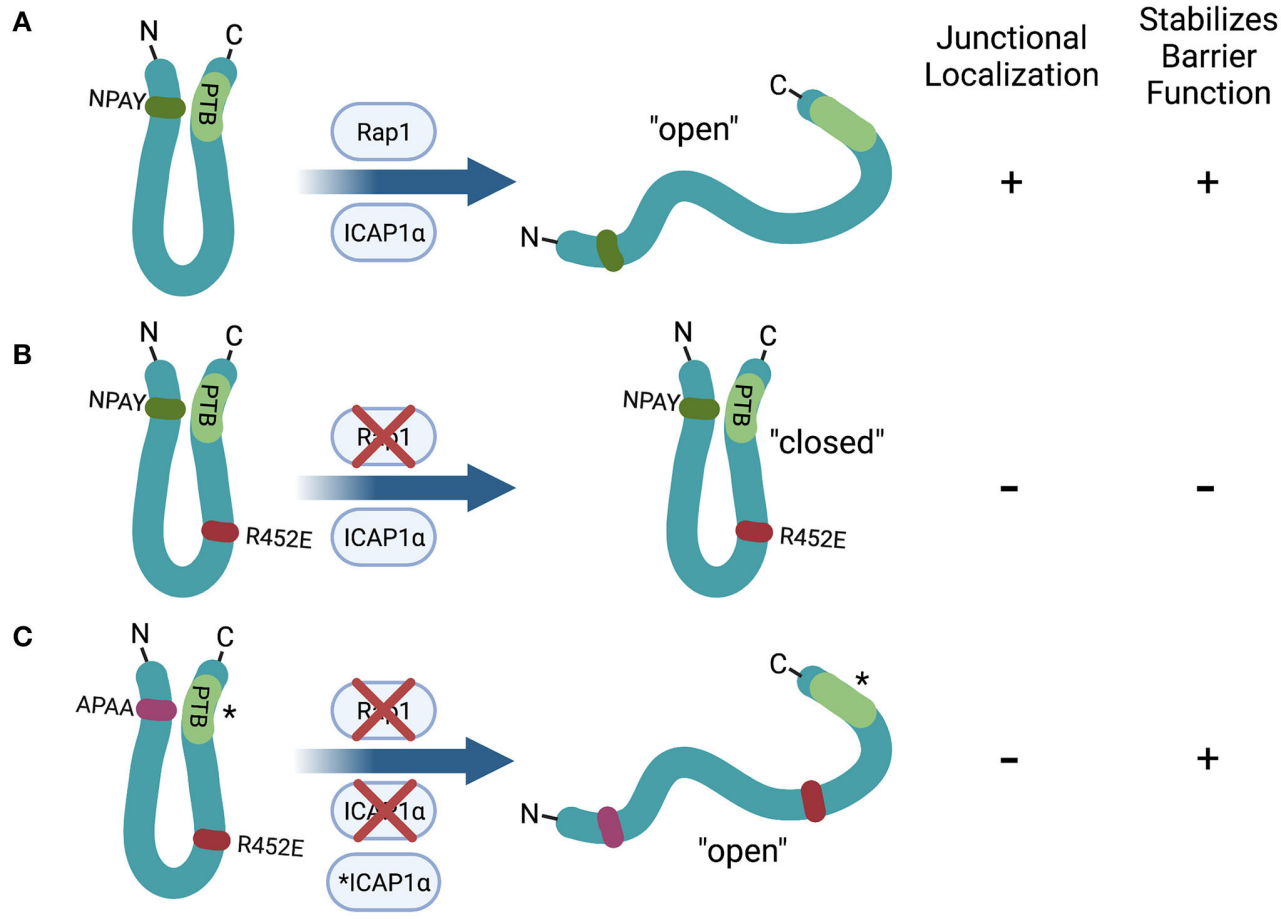
Hypothetical role of the N- to C- terminal self-interaction of KRIT1 in the regulation of barrier function. **(A)** Rap1 or ICAP1α binding to wildtype KRIT1 inhibits the N- to C-terminal interaction, leading to enhanced junctional localization (due to Rap1 binding) and stabilization of endothelial cell-cell contacts. **(B)** Mutation of the Rap1 binding interface (R452E) ablates junctional localization and barrier stabilization, likely due to a reduction in the proportion of the “open” conformation. **(C)** KRIT1 containing mutations in both the Rap1 binding interface (R452E) and the first NPXY motif (APAA) stabilizes cell junctions even though it remains cytoplasmic. Mutation of the PTB domain (*) has the same effect as mutating the NPXY motif, indicating that ICAP1α binding is not required for this effect. Created with Biorender.com.

In contrast, the ability of specific protein-protein interactions to regulate membrane localization of the CCM proteins, particularly KRIT1, has been more extensively studied. KRIT1 was first identified as an interacting partner of the small GTPase Rap1 in a yeast two-hybrid screen (11). Subsequent studies validated KRIT1 as a Rap1 effector that preferentially binds active (GTP-bound) Rap1 (24, 36, 37, 40). Binding of active Rap1 promotes the localization of KRIT1 to points of cell-cell contact where it associates with adherens junction proteins (24, 37), while co-expression of KRIT1 and RapGAP reduces the association of KRIT1 with β-catenin (24) (Figure 2). Binding of Rap1 to KRIT1 blocks the co-sedimentation of KRIT1 with microtubules, and reduces co-localization of KRIT1 with tubulin in baby hamster kidney (BHK) cells (40), suggesting that Rap1 activation could promote trafficking of KRIT1 from the cytoplasm to the plasma membrane. *In vitro* binding assays using KRIT1 peptide fragments initially revealed that Rap1 binds to the C-terminal FERM domain (24, 40, 42). One such study suggested a role for the F3 lobe of the FERM domain using yeast two-hybrid analysis (42). However, X-ray crystallography studies have definitively demonstrated that Rap1 binds KRIT1 at the interface of the F1 and F2 lobes (36). This supports prior reports that the KRIT1 FERM domain fragment could localize to adherens junctions, but mutation or deletion of the F1 lobe ablates this effect (24, 37). Furthermore, a charge switch mutation in this binding interface (R452E) results in a significant reduction in Rap1-binding affinity (37). As a result, KRIT1-R452E is unable to localize to adherens junctions. However, we recently reported that Rap1 binding, though a key regulator of KRIT1 junctional localization, was not absolutely required for the ability of KRIT1 to stabilize barrier function (58). In this study, we expressed various mutated forms of KRIT1 at replacement levels in KRIT1 shRNA expressing human pulmonary artery endothelial cells. Compared to wildtype KRIT1, KRIT1 containing a mutated Rap1 binding site (KRIT1-R452E) is unable to localize to adherens junctions and does not rescue barrier function of KRIT1 deficient cells. However, when we added an additional mutation of the first NPXY motif (APAA), which would block binding of ICAP1α or the N- to C-terminal self-interaction, we restored barrier function but not junctional localization. Furthermore, mutation of the KRIT1 PTB, which blocked the self-interaction but not ICAP1α association, also restored barrier function in the absence of junctional localization (58) (Figure 3). These data suggest that Rap1 binding may regulate KRIT1 in two distinct ways, first, it promotes junctional localization through an as yet undefined mechanism, and second, it negatively regulates the N- to C-terminal interaction, the latter of which appears critical for the function of KRIT1 and the CCM complex. This novel finding, while possibly controversial, may explain why the transmembrane protein HEG1 is not a necessary component of the CCM complex, despite the fact that it binds to KRIT1. HEG1 binds to KRIT1 at the interface of the F1 and F3 lobes of the KRIT1 FERM domain. Ablation of this interaction by mutation of KRIT1 (L717A,721A) disrupts localization of KRIT1 to endothelial junctions (39), suggesting that HEG1 may be important for anchoring KRIT1 at junctions (Figure 2). However, knockout of HEG1 *in vivo* failed to lead to the formation of CCM (59), suggesting that this binding interaction is dispensable for normal vascular development. Clearly, much remains to be understood about how the localization of the CCM complex to the plasma membrane, or more specifically cell-cell contacts, is regulated, which is critical to our ability to understand how this localization affects the functional outcome of CCM complex signaling.

### Cytoplasmic Localization of the CCM Complex

As mentioned above, the CCM proteins are often observed in the cytoplasm. KRIT1 can bind to microtubules, as demonstrated by co-sedimentation with tubulin in BHK fibroblast lysates. *In vitro* binding assays indicated that this interaction is mediated by regions in both the N- (residues 46–51) and C-termini (residues 569–572) of KRIT1. Binding of Rap1 or ICAP1α to KRIT1 inhibits KRIT1 binding to microtubules. Activation of Rap1 with non-hydrolysable GTPγS reduces co-sedimentation with tubulin. Similarly, over-expression of constitutively active Rap1 (RapV12) prevents co-localization of YFP-KRIT1 with fluorescently-tagged tubulin in cell culture (40). That Rap1 may regulate the interaction of KRIT1 with microtubules in some contexts was also supported by Liu et al. who showed that the KRIT1-R452E mutation reduces co-sedimentation with tubulin in human osteosarcoma epithelial cells [U2OS, (37)]. However, others have not observed co-localization of KRIT1 with the microtubule cytoskeleton in confluent human or bovine aortic endothelial cells (24, 58), which calls into question the ubiquity of this interaction. In addition, the microtubule binding domain in the N-terminus of KRIT1 overlaps with the reported nuclear localization sequence, thus studies to determine the relevance of the microtubule binding to KRIT1 function would be complicated by possible alteration of nucleocytoplasmic trafficking.

Another possible mediator of cytoplasmic localization of the CCM complex is the protein sorting nexin 17 (SNX17, Figure 2). Yeast two-hybrid screens as well as GST-trapping assays previously identified KRIT1 as an interacting partner of SNX17 and defined an interaction between the N-terminus of KRIT1 and the SNX17 FERM domain (60). Crystallography studies further confirmed this interaction, and pointed to the particular importance of KRIT1's second NPXY motif in this interaction (33) (Figure 1). Sorting nexins are involved in a variety of endocytic and endosomal processes, with SNX17 playing a role in endosomal recycling, particularly of integrins (61). Thus, it is possible that the presence of SNX17 on endosomal membranes could recruit the CCM complex away from the plasma membrane. Furthermore, based on the fact that both CCM2 and SNX17 bind to the second NPXY sequence on KRIT1, SNX17 could compete with CCM2 for binding to KRIT1, thus altering the composition of the signaling complex in a location specific manner. The implications of the interaction of KRIT1 with SNX17 are intriguing, and should be investigated further.

The interaction of KRIT1 with CCM2 has also been reported to promote cytoplasmic localization of both proteins. Zawistowski et al. first reported that co-expression of KRIT1 and CCM2 led to the cytoplasmic localization of both proteins in sub-confluent COS-7 cells. This paper also reported that ICAP1α can form a tertiary complex with KRIT1 and CCM2 (34). As is discussed in depth in the next section, ICAP1α expression promotes the nuclear localization of KRIT1. Thus together, these data suggest that the relative binding of ICAP1α and CCM2 to KRIT1 may control the distribution of the CCM complex. Indeed, Francalanci et al. found that the nuclear accumulation of ICAP1α and KRIT1 was lost in the presence of CCM2 (42), which could suggest that CCM2 binding retains KRIT1 in the cytoplasm or promotes nuclear export. CCM2 may also promote the cytoplasmic localization of CCM3, as over-expressed CCM3 exhibits more nuclear localization when the FAT domain is mutated and it no longer binds to CCM2 (50).

The cytoplasmic localization of KRIT1 could also be regulated by post-translational modification, such as phosphorylation. To this point, we recently demonstrated that activation of PKC, particularly PKCα, led to a predominantly cytoplasmic distribution of KRIT1 and blocked localization of KRIT1 to the nucleus in both sub-confluent and confluent endothelial cells. Pre-treatment with the antioxidant N-acetyl-cysteine reversed the ability of PKC activation to promote localization of KRIT1 to the cytoplasm, but did not go so far as to promote nuclear localization (62). Work is ongoing to determine the target(s) of PKCα which regulate KRIT1 nuclear-cytoplasmic shuttling, but these data raise the question of why the shuttling of KRIT1 (and potentially the CCM complex) between the cytoplasm and nucleus is so highly regulated, and what effect it might have on complex function or CCM pathogenesis.

### Nuclear Localization of the CCM Complex

Finally, the CCM proteins have been consistently observed in the nucleus (Figure 2). While CCM2 and CCM3 lack established nuclear localization or export sequences, KRIT1 has been reported to have both (41). Full-length KRIT1 partially localizes to the nucleus, as does the KRIT1 FERM domain [residues 409–736, (24)]. Truncating the FERM domain to eliminate the F1 subdomain eliminates this nuclear localization (24), as does mutating/deleting the F3 subdomain (42). Interestingly, compared to full-length KRIT1, a truncated KRIT1 construct containing the ankyrin repeats and the FERM domain (residues 207–736) is retained in the nucleus and is insensitive to PKC activation (62). These observations have led to the conjecture that KRIT1 contains two nuclear localization sequences, one in its N-terminus (residues 46-KKKRKK-51); and one in the C-terminus (residues 569-KKHK-572). Several studies have shown that mutation of the N-terminal KRIT1 NLS is sufficient to decrease localization of KRIT1 to the nucleus (42, 43), while mutation of the second NLS in full-length KRIT1 is insufficient (43), suggesting that the N-terminal NLS is functionally dominant and that the nuclear localization of the FERM domain may be driven by some other mechanism. Complicating matters, an N-terminal fragment (residues 1–207), though it contains the NLS, was shown to have a cytoplasmic distribution in transfected HeLa cells (42).

Recently, Draheim et al. reported that KRIT1 nuclear localization can be driven by its interaction with ICAP1α, even in the absence of the KRIT1 NLS. Mutation of either ICAP1α's NLS or KRIT1's ICAP1α binding site significantly inhibited KRIT1 nuclear localization (43). This agrees with early reports that demonstrated that co-expression of exogenous KRIT1 and ICAP1α in COS-7 cells induced the complete nuclear localization of both proteins (41). ICAP1α binding to KRIT1's first NPXY motif also sterically hinders KRIT1's association with microtubules, similar to what has been suggested of the KRIT1∙Rap1 interaction (40). Interestingly, the sequence through which KRIT1 likely binds to microtubules is the same stretch of lysines that form the KRIT1 NLS. Thus, changes in the accessibility of this sequence, whether through ICAP1α binding or through Rap1 binding, could cause dissociation from microtubules and also allow for nuclear localization.

While the mechanism(s) governing trafficking of KRIT1 into the nucleus appear clear, the shuttling of KRIT1 out of the nucleus is much less well understood. Sequence prediction has led to several papers proposing that KRIT1 has a nuclear export sequence in the C-terminal FERM domain [residues 551–559, (42, 44)]. However, when this sequence was mutated in a recent study, it failed to lead to enrichment of KRIT1 in the nucleus (43), suggesting that it is not a functional NES. Our recent study suggests that export could be regulated by PKC activity (62), but additional work will be necessary to fully characterize the mechanism. Intriguingly, Zhang et al. reported in 2007 that treatment with leptomycin B, an inhibitor of exportin 1, led to accumulation of KRIT1 and CCM2 in the nucleus (44), leaving the possibility open that, if KRIT1 does not contain a functional NES, perhaps CCM2 does.

In sum, it is clear that localization of the CCM proteins is dynamically regulated by several potential mechanisms. The current models of CCM signaling in the literature alternatively obfuscate where in the cell the CCM complex is active (though they restrict the possibilities to cytoplasm or membrane) or point to membrane localization as being important- and imply that cytoplasmic localization of the CCM complex acts to sequester the complex away from the plasma membrane. The available evidence supports both models, as we lack the direct evidence needed to resolve these possibilities. However, in order to fully understand the link between loss of function mutations in the CCM proteins and CCM pathogenesis, future work will need to address this gap in knowledge and illuminate how these key binding interactions are segregated in time and place, and how they collectively determine the location and function of the CCM complex.

## Role of Subcellular Localization in Regulating the Signaling Downstream of the CCM Complex

### At the Membrane: Adherens Junctions, Tight Junctions, and Integrins

The early finding that KRIT1 localizes to points of cell-cell contact (24) led to the development of a broadly accepted working model in which junctional localization of the CCM signaling complex is required to maintain endothelial and vascular homeostasis. Conversely, loss of junctional localization of KRIT1, such as after treatment with thrombin, correlated with down-regulation of VE-cadherin adhesion and loss of barrier function (24). Conspicuously, most of the studies supporting this concept have focused on KRIT1, though CCM2 and CCM3 are assumed to co-localize with KRIT1 in order to form a functional signaling complex. In confluent endothelial cells, KRIT1 co-immunoprecipitates with the integral adherens junction proteins β-catenin and p120-catenin (24), and stabilizes the interaction of β-catenin with VE-cadherin (63), a classical indication of mature adherens junctions (64). Additionally, CCM lesions from both human patients and mouse models exhibit a reduction in TJ protein expression (4, 65, 66). In particular, claudin-5, the major claudin isoform in endothelial cells, is downregulated after loss of KRIT1 (65, 66). While the mechanism by which loss of CCM protein expression leads to reduced barrier function remains undefined, KRIT1 appears to affect cell-cell contacts by stabilizing β-catenin association with adherens junction complexes. Accordingly, loss of KRIT1 expression induces phosphorylation of β-catenin at Y654, a key residue regulating the cadherin∙β-catenin interaction (67), leading to translocation of β-catenin to the nucleus and changes in β-catenin mediated TCF/LEF transcriptional activity, including increased expression of cyclinD1 and *Vegf-a* *(63)*. Claudin-5 transcription is also under the control of a β-catenin∙FoxO1∙Tcf4 repressor complex, thus increased β-catenin signaling in the nucleus negatively regulates claudin-5 gene transcription (68). Though the total effect of increased β-catenin transcriptional activity on CCM pathogenesis has not been examined, Distefano et al. demonstrated that increased expression of VEGF in KRIT1 deficient endothelial cells formed a feed-forward mechanism that promoted several CCM-related changes in endothelial phenotype (63). Furthermore, blocking the activation of the VEGF receptor VEGFR2 limited lesion formation and bleeding in a mouse model of CCM (69), suggesting that down-regulation of the β-catenin∙VE-cadherin complex may be a critical signal in CCM pathogenesis.

Another potential mechanism reliant on the localization of CCM proteins to cell-cell contacts lies downstream of HEG. Mutating the binding sites for HEG1 or Rap1 on KRIT1 inhibits KRIT1 localization to endothelial junctions and disrupts junctional VE-cadherin (39). Additionally, *in vitro* binding and immunofluorescence data indicate that the Rap1 effector Rasip1 also appears to be anchored at cell-cell contacts by HEG1 (70), suggesting that HEG1 is an important focus for Rap1 signaling. The Rap1∙Rasip1 interaction appears to inhibit Rho signaling through activation of the RhoGAP ArhGAP29 (71). Though HEG1 does not regulate the Rap1∙Rasip1 or Rasip1∙ArhGAP29 interactions, because KRIT1 and Rasip1 both bind HEG1 at cell-cell contacts (70), HEG1 may be an important center point for regulation of a balance between Rap1 and RhoA signaling necessary for junctional homeostasis. Interestingly, Castro et al. reported that postnatal deletion of Cdc42, a downstream target of Rap1 signaling, also leads to formation of CCM-like lesions (18). This further suggests that CCM pathogenesis may be linked to activation/inactivation of specific signals downstream of Rap1 signaling.

KRIT1 also plays a role in regulating β1-integrin activity through its interaction with ICAP1α (Figure 2). KRIT1 competes with the β1-integrin cytoplasmic domain to bind ICAP1α, and can promote β1-integrin activation (32, 43). However, recent examination of these signaling mechanisms by Lisowska et al. suggests that KRIT1 or CCM2 depletion triggers enhanced development of centrally localized β1-integrin-dependent focal adhesions (72), which runs contrary to expectations based on a competitive mechanism. This study also found that activation of β1-integrin correlated with increased RhoA signaling and remodeling of fibronectin ECM structure after loss of either KRIT1 or CCM2 (72). The finding that loss of KRIT1 or CCM2 upregulates β1 integrin activity corroborates previous work by Faurobert and colleagues, who proposed that KRIT1 depletion in HUVEC destabilized the ICAP1α protein leading to ICAP1α degradation and subsequent increased β1-integrin activation (73). The contradictory results may be explained by the observation that the EA.hy926 cell line used in the studies which demonstrated competitive inhibition of the ICAP1α∙integrin interaction by KRIT1 express more ICAP1α and KRIT1, but significantly less β1-integrin compared to HUVECs (32, 73).

Another potential explanation for increased β1-integrin activation after KRIT1 depletion may lie in changes in Rap1 signaling. Studies have established that Rap1 is major regulator of integrin activation, particularly β1-integrin (74), likely *via* interaction with the integrin-activating protein talin (75–77). Following this line of thought, it is possible that depletion of KRIT1 would free Rap1 to bind to other effectors. Excess free Rap1 could then promote talin-mediated activation of β1-integrin, leading to the development of focal adhesions, stress fibers, and other phenotypes associated with β1-integrin activation.

As described earlier in this review, our recent study challenges the idea that the physical localization of KRIT1 at the plasma membrane is required for cell contact stability. Mutant KRIT1 in which the Rap1 binding domain is disrupted and in which the N- to C-terminal interaction is blocked by mutation of the first NPXY motif or the PTB domain rescues β-catenin localization and restores barrier function of KRIT1-depleted endothelial cells. Notably, these mutants have a predominantly cytoplasmic localization, and are not present at cell-cell contacts nor at the basal membrane. Thus, membrane localization appears dispensable for the ability of KRIT1 to stabilize endothelial cell-cell contacts (Figure 3). Interestingly, we found that stabilization of cell-cell contacts does correlate with the capacity of KRIT1 to regulate integrin signaling, and specifically to limit β1 integrin activation. As previously reported, we observed that loss of KRIT1 increased β1 integrin activity. This increase could be rescued by expression of wildtype KRIT1 or KRIT1 in which the N- to C-terminal self-interaction was ablated by mutation of either the first NPXY motif or the PTB domain. However, KRIT1 containing the R452E mutation failed to reverse the activation of β1 integrin and cells expressing this construct exhibited large centralized β1-dependent focal adhesions similar to KRIT1 shRNA alone (58). Thus, these data suggest a potential connection between the regulation of cell-cell contact and cell-matrix contact by the CCM complex that should be explored further.

### In the Cytoplasm: Kinase Cascades

The effect of cytoplasmic localization on the function of the CCM complex has not yet been extensively tested. However, the CCM proteins, particularly CCM2 and CCM3, bind to several protein partners with a presumed cytoplasmic distribution. CCM2 binds to MKK3 and MEKK3, which regulate activation of p38 MAPK in response to stress and inhibit BMK1/ERK5 activation respectively (26, 48). CCM3 binds to the STRIPAK complex, which is found in the cytoplasm and at the membrane and has several functions, including regulation of cell polarity and Golgi assembly (51) (Figure 2). While the interaction of CCM2 and CCM3 with these larger complexes has been well documented, it is unknown whether the organization or function of these complexes is affected by specific subcellular localization of the CCM proteins. Precedent for such regulation exists, as there are many examples of scaffolding proteins regulating MAPK signaling cascades, including the classic scaffolds Ste5 and KSR which control MAPK pathway localization (i.e., membrane anchoring) and signaling efficiency (78). In this manner, the CCM complex could target or anchor these signaling complexes to the appropriate cellular location to receive incoming signals, and/or control the flow of signaling information to specific downstream processes.

### In the Nucleus: A Blank Page

Lastly, despite the widespread presence of KRIT1, CCM2 and CCM3 in the nucleus, only one publication has investigated a possible function for the CCM proteins in the nucleus. Using ultrastructural immunocytochemistry, Marzo et al. showed that KRIT1 localized to perichromatin fibrils, which are markers of transcriptional activity (79), as well as to the dense fibrillar component of the nucleolus which contains pre-ribosomal RNA (80), which hints at a possible role in transcriptional regulation. This, combined with the presence of a Nudix domain in KRIT1, makes it tempting to hypothesize that KRIT1 could bind directly to nucleic acids and regulate transcription or RNA stability, as do several of members of the Nudix protein superfamily (81). This could provide another mechanistic link between expression of the CCM proteins and changes in gene expression, which have been widely reported (22, 82). In addition, it has been proposed that KRIT1 and ICAP1α regulate each other by sequestering the other partner inside the nucleus, thus preventing interaction with cytoplasmic or membrane proteins. This idea is supported by the positive influence that the KRIT1 NLS exerts on ICAP1α nuclear localization (43), which would theoretically diminish the ability of ICAP1α to suppress β1 integrin activation. Accordingly, one could propose several mechanisms by which the localization of the CCM proteins in the nucleus could regulate CCM complex function, however it is still unclear whether, and how, this would occur.

## Discussion

Loss of function mutations in KRIT1, CCM2 or CCM3 lead to the development of CCM, a process that has been shown to involve major changes in endothelial function and behavior. The CCM proteins suppress cell division and inflammatory signaling by regulating the p38-MEKK3-KLF2/4 signaling axis (26, 83–85) while also regulating oxidative stress responses (22, 86–89), autophagy (23), apoptosis (87) and cell contractility (54, 72) (in addition to stabilizing cell-cell contacts). However, most of these disease-mediating mechanisms have only been tied to the expression of the CCM proteins, not to their localization or function. Thus, how CCM protein localization fits in the context of CCM pathology is unclear. What's more, many, if not the majority, of the CCM-causing mutations described in the literature are nonsense mutations which lead to premature termination of translation (90, 91). CCM may develop as the result of nonsense-mediated mRNA decay of CCM protein transcripts (91–93) or due to degradation of the truncated protein products *via* the unfolded protein response. This implies that CCM develops due to the complete lack of expression of one CCM protein, rather than the presence of non-functional, truncated proteins. However, as the three CCM proteins form a tripartite complex (52, 94), loss of one CCM protein could result in perturbation of the localization and function of the remaining complex members, which is indeed the case. This could eventually explain why, for example, patients with CCM3 mutations display earlier and more severe disease (30). By continuing to advance our knowledge of the mechanisms regulating the individual CCM proteins and the CCM signaling complex, we can not only discover more about the mechanisms that underlie CCM pathogenesis, but potentially identify new therapeutic targets and perhaps expand our understanding of other endothelial pathologies.

At the risk of sounding like a broken record, it is clear that much work remains to be done in order to fully understand how the CCM complex is regulated, whether by binding interactions, subcellular localization, or other mechanisms. Current knowledge is not only incomplete, but complicated by differences in cell type, cell density, and expression level between studies, making it difficult to form solid conclusions. This is a critical need, as only by being able to fully understand and manipulate the components and interactions of the CCM complex will we be able to answer such questions as: what is the function of the CCM complex in the nucleus, does the CCM complex generate differential downstream signals depending on its location, and how does loss of just one CCM complex protein lead to the development of CCM? This will require both a fuller understanding of the CCM interactome as well as cutting-edge approaches to track protein location and binding (potentially in real time). To make these future studies the most effective, it will be important to consider effects of the level of protein expression (i.e., over-expression vs. replacement studies), as well as issues caused by differences in cell type (i.e., epithelial vs. endothelial) and cell culture conditions (i.e., sub-confluent vs. confluent). The recent interest in structure-function relationships, particularly in regard to KRIT1, is encouraging, but we still know relatively little about these relationships in CCM2 and CCM3. These gaps in knowledge will need to be filled if we are to someday understand how disrupting the balance of protein-protein interactions in the greater CCM complex (either by mutation, manipulating expression, or post-translational modification) contribute to endothelial dysfunction and CCM pathology.

## Author Contributions

HS and AG both contributed to the conceptual development and writing of this manuscript. All authors contributed to the article and approved the submitted version.

## Funding

This work was supported by grants from the National Institutes of Health (HL117885 and HL141131 to AG) and the State of New York (FuzeHub New York State Technology & Innovation Grant to AG).

## Conflict of Interest

The authors declare that the research was conducted in the absence of any commercial or financial relationships that could be construed as a potential conflict of interest.

## Publisher's Note

All claims expressed in this article are solely those of the authors and do not necessarily represent those of their affiliated organizations, or those of the publisher, the editors and the reviewers. Any product that may be evaluated in this article, or claim that may be made by its manufacturer, is not guaranteed or endorsed by the publisher.
